# Risk profiling of tobacco epidemic and estimated number of smokers living in China: a cross-sectional study based on PBICR

**DOI:** 10.1186/s12889-024-18559-x

**Published:** 2024-08-15

**Authors:** Siyuan Liu, Haozheng Zhou, Wenjun He, Jiao Yang, Xuanhao Yin, Sufelia Shalayiding, Na Ren, Yan Zhou, Xinyi Rao, Nuofan Zhang, Man Xiong, Yueying Wang, Wenfu Yang, Yibo Wu, Jiangyun Chen

**Affiliations:** 1 School of Public Health, Southern Medical University, Guangzhou510515, Guangzhou510515, China; 2 School of Health Management, Southern Medical University, Guangzhou510515, China; 3 Institute of Chinese Medical Sciences, University of Macau, Macau, China; 4 Operation Management Department, Zhuhai People’s Hospital (Zhuhai Hospital Affiliated with Jinan University), Zhuhai, China; 5 School of Public Heath, Peking University, Beijing100091, China

**Keywords:** Smoking prevalence, China, Spatial inequality, Bayesian geostatistical models

## Abstract

**Background:**

Evidence on the prevalence of smoking in China remains insufficient, with most previous studies focusing on a single region. However, smoking prevalence exhibits significant inequalities across the entire country. This study aimed to evaluate the risk of tobacco prevalence across the country, taking into account spatial inequalities.

**Methods:**

The data used in this study were collected in 23 provinces, 5 autonomous regions, and 4 municipalities directly under the central government in 2022. Large population survey data were used, and a Bayesian geostatistical model was employed to investigate smoking prevalence rates across multiple spatial domains.

**Findings:**

Significant spatial variations were observed in smokers and exposure to secondhand smoke across China. Higher levels of smokers and secondhand smoke exposure were observed in western and northeastern regions. Additionally, the autonomous region of Tibet, Shanghai municipality, and Yunnan province had the highest prevalence of smokers, while Tibet, Qinghai province, and Yunnan province had the highest prevalence of exposure to secondhand smoke.

**Conclusion:**

We have developed a model-based, high-resolution nationwide assessment of smoking risks and employed rigorous Bayesian geostatistical models to help visualize smoking prevalence predictions. These prediction maps provide estimates of the geographical distribution of smoking, which will serve as strong evidence for the formulation and implementation of smoking cessation policies.

**Highlights:**

Our study investigated the prevalence of smokers and exposure to secondhand smoke in different spatial areas of China and explored various factors influencing the smoking prevalence. For the first time, our study applied Bayesian geostatistical modeling to generate a risk prediction map of smoking prevalence, which provides a more intuitive and clear understanding of the spatial disparities in smoking prevalence across different geographical regions, economic levels, and development status. We found significant spatial variations in smokers and secondhand smoke exposure in China, with higher rates in the western and northeastern regions.

**Supplementary Information:**

The online version contains supplementary material available at 10.1186/s12889-024-18559-x.

## Introduction

Tobacco prevalence poses significant threats to both human health and the economy. Smoking is estimated to cause the death of eight million people globally each year [1]. Predictions suggest that by 2030, this number will rise to 10 million globally[2, 3]. Currently, the prevalence of tobacco consumption is shifting from high-income to middle-and-low-income countries globally. China, as the world’s largest developing country and tobacco producer and consumer, produces 43% of the world’s tobacco[4]. It is estimated that the total number of deaths due to smoking in developing countries, including China, will reach 70% [3], presenting a severe problem requiring urgent attention. China, with its smoking population accounting for 38% of the global total, is estimated to have over 350 million smokers[5]. This massive number of smokers has contributed greatly to the increasing rate of smoking prevalence in China[6]. The above findings demonstrate that contemporary China continues to face significant risks associated with smoking, making it the most prominent preventable risk factor for adverse health outcomes in the country. Therefore, tobacco control remains a top priority for public health and safety in China, and it will have significant implications for global tobacco control efforts[7].

Within the context of tobacco control measures in China, gaining a thorough understanding of the distribution of tobacco use prevalence is a prerequisite for guiding and formulating effective tobacco control policies to curb the prevalence of tobacco use in China. Previous studies have largely focused on surveys of smoking prevalence in specific regions within China, revealing significant correlations between smoking rates and social, health, environmental, and economic inequalities, which can also have a major impact on smoking cessation strategies[8–13]. However, most of these studies are limited to specific regions and spaces, which may affect the reliability and generalizability of their conclusions. Only a few nationwide surveys have described the distribution of smoking prevalence among provinces and populations[14–16]. However, this existing research has not provided a clear geographic distribution of tobacco use and a mapped risk of smoking prevalence, which may further prevent governments and policymakers from developing more targeted interventions to aid smoking cessation programs.

Indeed, understanding spatial disparities and regional inequalities is crucial for controlling smoking rates. This knowledge can assist the government in formulating region-specific tobacco control strategies and policies tailored to different areas in China. It can effectively facilitate comprehensive coverage of smoking control measures throughout the country. The Bayesian geostatistical model is a robust and commonly used approach that integrates disease survey data with underlying risk factors to generate disease risk maps. It is also the most widely employed method in the field of risk prediction, enabling us to forecast the infection risk in unobserved areas[17]. This study will use a Bayesian geostatistical model to conduct a large-scale survey covering a wide range of factors and based on the differences in smoking prevalence across multiple spatial and regional settings, to predict the risk of smoking prevalence in various regions of China. At the same time, the remaining data can provide reference for analyzing the relationship between individual factors and smoking, as a supplement to the main results. The findings from our study will guide to improve the formulation of national smoking cessation intervention policies.

## Materials and methods

### Study design

This study is a large-scale population survey conducted in China, aimed at better understanding spatial disparities and inequality among regions. The study aims to fill this gap by investigating differences in smoking prevalence rates across multiple spatial settings and providing guidance for the implementation of a national smoking cessation strategy.

The data utilized in this study were derived from the PBICR survey, conducted in 2022 across 23 provinces, 5 autonomous regions, and 4 direct-administered municipalities in China. The survey, led by the School of Public Health at Peking University, has been officially registered with the China Clinical Trial Registry under the registration number ChiCTR2200061046. Employing a multi-stage sampling strategy with a random digit table method, the survey selected 2–6 cities from each non-capital administrative region of the provinces and autonomous regions, resulting in a total of 120 cities.

To the results of the “Seventh National Population Census of China in 2021,” samples were selected within each city based on gender, age, and urban-rural distribution attributes, utilizing a quota sampling approach to ensure the demographic representation of the samples in terms of gender, age, and urban-rural distribution. Probability sampling and equal probability sampling (also known as stratified sampling) were employed at different levels, ranging from the community/village level to the individual level.

In the end, a total of 21,916 individuals were included in this survey, with a minimum sample size of 500–2500 individuals per province (autonomous region/municipality). The response rate was 71.8%, and the qualification rate was 96.8%. At least one interviewer or interview team was recruited in each city, with each interviewer responsible for collecting 30–90 questionnaires, and each team tasked with collecting 100–200 questionnaires. Interviewers distributed questionnaires to all participants through the online survey platform (https://www.wjx.cn/), obtained informed consent from each participant, and recorded the questionnaire number assigned to them. Participants were residents of China aged 12 and above, voluntarily consenting to participate, with individuals experiencing mental or cognitive impairments excluded from the study[18] (Supplementary Fig. 1).

### Study population

 To analyze the data, we included participants who met the following criteria: (1) Participants aged 12 and over; (2) Chinese nationals; (3) permanent residents of China (with an annual travel time of not more than 1 month); (4) able to complete the online questionnaire independently or with the assistance of the interviewer; (5) able to understand the meaning of each item in the questionnaire; (6) individuals who are unable to provide accurate responses due to known mental illness or psychiatric disorders; and (7) not participating in other activities (Supplementary Fig. 1).

### The variables included in the construction of the bayesian geostatistical model

Climate, demographic, and environmental data were derived from readily available sources, as summarized in Table 1. The land surface temperature (LST) for 2013 was averaged. In addition, five socioeconomic data were obtained from the Socioeconomic Data and Applications Center, including the Human Influence Index (HII), infant mortality rate (IMR), global human built-up and settlement extent (HBASE), annual PM2.5, global human modification of terrestrial systems (Terrestrial). Data on night lights and elevation were also obtained. We linked the survey locations with missing data to the values at their nearest pixel. The survey data aggregated by areas were associated with the mean value of covariates within each area and georeferenced using the corresponding centroid.

**Table 1:** Remote sensing data sources employed^a^

Source	Data type	Data period	Temporal resolution	Spatial resolution
SEDAC/Terra^b^	LST^f^	2013	8 days	1 km
SEDAC/DMSP^c^	Night Light	2013	-	1 km
WorldClim^d^	Elevation	2000	-	1 km
SEDAC^e^	HII^g^	1995–2004	-	1 km
SEDAC^e^	IMR^h^	2015	-	1 km
SEDAC^e^	HBASE^i^	2010	-	30 m
SEDAC^e^	Annual PM2.5	2019	1998–2019	0.01degree
SEDAC^e^	Terrestrial^j^	2016	-	1 km

^a^All data were accessed on December 1, 2022

^b^Moderate Resolution Imaging Spectroradiometer (MODIS)/Terra; available at: http://modis.gsfc.nasa.gov/

^c^Defense Meteorological Satellite Program (DMSP); available at: https://sedac.ciesin.columbia.edu/data/set/sdei-viirs-dmsp-dlight

^d^Available at: http://www.worldclim.org/current

^e^Socioeconomic Data and Applications Center; available at: http://sedac.ciesin.org/

^f^Land surface temperature (LST) day and night

^g^Human influence index

^h^Infant Mortality Rate

^i^Global Human Built-up And Settlement Extent

^j^The Global Human Modification of Terrestrial Systems

### Assessment of outcome variables

The outcome variables included in this study are smokers and secondhand smoke exposure. The definition of smokers was based on the question ‘Do you have a smoking habit?’ If the response was ‘Yes, regular cigarettes,’ ‘Yes, e-cigarettes,’ or ‘Yes, both,’ the variable would be categorized as smokers. If the response was ‘Former smoker (quit smoking)’ or ‘No,’ the variable would be defined as non-smoking.

In addition, secondhand smoke exposure was jointly determined by the combined responses to the questions ‘Who around you has a smoking habit?’ and ‘Do the mentioned smokers smoke in your presence?’ If the answer to the former was ‘Yes’ and the answer to the latter was ‘Yes,’ the individual would be defined as secondhand smoke exposure. If the response to the former was ‘Yes’ and the response to the latter was ‘No,’ or the response to the former was a direct ‘No’, the individual would be defined as non-exposed to smoke (Supplementary Table 1).

### Assessment of covariates

The covariates include the smoker’s gender, age, ethnicity, religion, family’s monthly per capita income, level of education, occupation, permanent residence, marital status, and social status (Supplementary Table 2).

### Variable selection and geostatistical modeling

The variables selected from Bayesian variable selection are listed in Supplementary Table 3. In the final geostatistical regression model, both smokers and secondhand smoke exposure are negatively associated with Night light and PM2.5 at the national level. Night light represents an index of the economic level in the region, with higher values indicating better economic conditions. PM2.5 reflects some differences related to rural-urban areas, work environments, etc., and lower PM2.5 values in a locality may suggest that the area is relatively underdeveloped. Spatial variance and non-spatial variance represent the variability of the map in spatial and non-spatial dimensions.

Studies have indicated a significant correlation between average elevation and economic status in China. The average elevation in China gradually increases from east to west, coinciding with a decrease in economic development from east to west. Consequently, elevation can reflect the economic development imbalance across eastern, central, and western regions in China[19]. This phenomenon is closely linked to local tobacco consumption patterns.

Our studies involve downloading relevant datasets from publicly accessible remote sensing databases, including MODIS, DMSP, and the Social and Economic Data and Applications Center. Therefore, the variables in Table 1 are derived entirely from remote sensing data. Land Surface Temperature (LST) data are sourced from SEDAC and the Moderate Resolution Imaging Spectroradiometer (MODIS) on the NASA Terra satellite. Nighttime light data are obtained from DMSP, managed by SEDAC. Elevation data come from the global climate database of WorldClim. An extensive literature review reveals that studies conducted by Kilcommons LM et al. in 2023 explored the relationship between outdoor nighttime light and prostate cancer using DMSP data[20]. Similarly, Sun C et al. investigated the spatiotemporal dynamics of carbon emissions in the Yangtze River Delta region of China[21], and Li Y et al. predicted the elderly dependency ratio in China[22], all of which are closely tied to urbanization. Furthermore, these studies emphasized the advantages of DMSP nighttime light data over conventional sensors, citing its broad coverage, rapid update frequency, and comprehensive reflection of human activities and regional economies. It is important to note that all datasets were accessed on December 1, 2022, ensuring the utilization of the latest available data at that time.

We have also incorporated other crucial datasets, such as the Human Influence Index (HII), Infant Mortality Rate (IMR), and Global Human Built-up and Settlement Extent (HBASE), all sourced from SEDAC. These datasets have been widely utilized in various studies, ensuring the stability and reliability of our results. For instance, Kilcommons LM et al. (2023) investigated the relationship between outdoor nighttime light and prostate cancer using DMSP data[20]. Sun C et al. explored the spatiotemporal dynamics of carbon emissions in the Yangtze River Delta region of China. Additionally[21], Li Y et al. predicted the elderly dependency ratio in China[22]. These applications further validate the robustness of our findings.

### The bayesian spatial logistic regression model

Bayesian geostatistical regression models were constructed to evaluate the relationship between various influencing factors (covariates) and both smokers and exposure to secondhand smoke. For the ${ i}^{th}$ individual belonging to the ${ j}^{th}$ position, we assumed a smoking status ${Y}_{ij}$ based on the Bernoulli distribution (${Y}_{ij}$ = 1 for smokers/existence of secondhand smoke exposure, and ${Y}_{ij}$ = 0 for non-smokers/non-existence of secondhand smoke exposure), where ${p}_{ij}$ denotes the prevalence of smokers/exposure to secondhand smoke:

$$Y_{ij}\;\sim\;Bernoulli\;\left(p_{ij}\right)$$

Here, the logit transform of ${p}_{ij}$ is assigned to a linear combination of effects:

$$logit\left({p}_{ij}\right)={\beta }_{0}+{\varvec{X}}_{ij}^{T}\varvec{\beta }+{\phi }_{j}+{\omega }_{j}$$

In which, ${\varvec{X}}_{ij}$, ${\beta }_{0}$, and $\varvec{\beta }$ are respectively represented as the covariate matrix, intercept, and regression coefficient vector. These constitute the fixed effects (level 1) of the model. A spatial random effect ${\phi }_{j}$ is introduced in the model to account for variations at specific locations. It is assumed to follow a zero-mean Gaussian process, with its covariance defined as the spatial covariance matrix ${K}_{s}$:

$$\varphi\sim GP\;\left(0\;,\;K_s\right)$$

The spatial covariance matrix K_s is defined as a stationary Matérn covariance function, given by ${K}_{s}=\frac{{\sigma }^{2}}{{2}^{v-1}\varGamma \left(v\right)}{\left(\kappa D\right)}^{v}{K}_{v}\left(\kappa D\right)$, ${\sigma }^{2}=\frac{1}{4{\uppi }{{\upkappa }}^{2v}{\tau }^{2}}$. Here, $D$ represents the Euclidean distance matrix, ${K}_{v}$ denotes the modified Bessel function of the second kind, with the smoothness parameter $v$ fixed at 1, $\kappa$ as the scale parameter within the range $r$ = $\sqrt{8v}/\kappa$, and $\tau$ as the precision parameter for the spatial variance ${\sigma }^{2}$. ${\omega }_{j}$represents the unstructured random effect, assumed to follow a zero-mean normal distribution:

$$\omega_j\sim Normal\;\left(0,\delta^2\right)$$

It describes the unexplained random variance, distinct from the variance explained by the fixed-effect predictor factors ${\omega }_{j}$ constitutes the random effects of the model (level 2).

### Statistical analysis

We used the Bayesian spatial geographic statistics method to fit the model with remote sensing and social demography spatial distribution data, to estimate the risks of smokers and secondhand smoke exposure nationwide.

Supplementary Table 4 elaborates on the selection of the Bayesian models, where different variable combinations correspond to different models, and the model selection is based on the principle of minimum DIC. It can be found that the models with the Night light and PM2.5 variables have the minimum DIC for smokers and secondhand smoke exposure, which are 17509.2 and 27862.73, respectively. Therefore, the selected Bayesian models for both smokers and secondhand smoke exposure include Night light and PM2.5 variables.

To address the issue of model collinearity, we performed a Pearson correlation analysis. In the correlation analysis, two variables with a correlation coefficient greater than 0.8 cannot be included in the regression model simultaneously to avoid multicollinearity (Supplementary Fig. 2).

The model was fitted on a randomly selected subset of 80% of the survey locations and validated on the remaining 20%, comparing the observed and predicted disease prevalence values using the Bayesian credible intervals (BCI) of the various probability coverages of the prediction distribution and the area under the curve (AUC) of the receiver operating characteristic (ROC) curve obtained from the validation dataset at the individual level[23], where the AUC ranges from 0.5 to 0.7 indicating poor discrimination ability, 0.7–0.9 indicating reasonable capacity[24], and > 0.9 indicating good capacity (Supplementary Fig. 3). Moreover, we covered the study area with a 10 × 10 km grid and used Bayesian geospatial statistics to predict the prevalence risks of smokers and secondhand smoke exposure on the centroids of grid pixels. To evaluate the performance of the method, we calculated the AUC of the ROC curve based on the predicted and observed overall disease prevalence, and the percentage of observed values included in the 95% BCI of the prediction distribution.

By overlaying the pixel-based prevalence risks on population grids, the number of smoking individuals was estimated for each pixel, then summed up within the country and divided by the country’s population to estimate the adjusted smoking prevalence rates by province, city, and rural area.

Using integrated nested Laplacian Approximation (INLA) to compute Bayesian geostatistical models, INLA has been developed as a computational efficiency alternative to MCMC 70. The R-INLA software package was used for spatial analysis in R software, and the predicted values were the median of the posterior distribution. Meanwhile, other demographic data were cleaned and analyzed by SAS V9.4 and R software.

## Results

### Multifactorial analysis

**Fig. 1:**
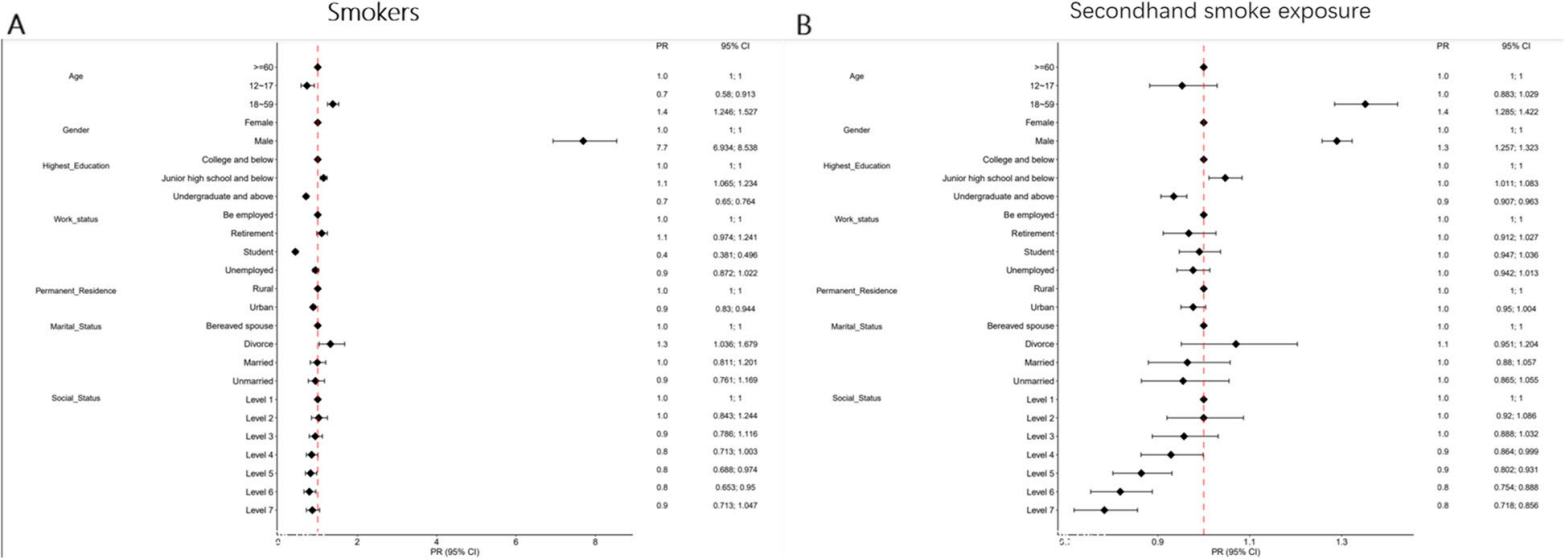
Smokers and secondhand smoke exposure

The basic characteristics of smokers and smoke-exposed individuals are presented in the univariate analysis (Supplementary Table 5). Multifactorial analysis (Figure 1) shows that for smokers, age is a significant factor, with the age group of 12–17 years showing a protective effect compared to those aged 60 years and above (PR:0.7, 95%CI:0.58–0.913), and the age group of 18–59 years showing a risk factor (PR:1.4, 95%CI:1.246–1.527). Gender is also a significant factor, with males being a risk factor compared to females (PR:7.7, 95%CI:6.934–8.538). In terms of educational level, those with a primary school education or lower were found to be at higher risk (PR:1.1, 95%CI:1.065–1.234) compared to those with a college degree or higher, who showed a protective effect (PR:0.7, 95%CI:0.65–0.764). Employment status did not show a significant difference in rates of smokers compared to those who were employed, with retired or unemployed individuals showing similar rates. However, students were found to have a protective factor (PR:0.4, 95%CI:0.381–0.496). In terms of residential location, urban residents showed a protective effect (PR:0.9, 95%CI:0.83–0.944) compared to rural residents. Regarding marital status, divorce was found to have a protective effect (PR:1.3, 95%CI:1.036–1.679) compared to widowhood. Finally, in terms of social status, only levels 5 (PR:0.8, 95%CI:0.688–0.974) and 6 (PR:0.8, 95%CI:0.653–0.95) showed a protective effect compared to level 1, with no significant difference in rates of smokers observed for the other levels.

Based on the forest plot of secondhand smoke exposure, the age group of 18–59 years had a higher risk of secondhand smoke exposure compared to those aged 60 years and above (PR:1.4, 95%CI:1.285 ~ 1.422), while those aged 12–17 years had no significant difference in secondhand smoke exposure rate compared to the reference group. In terms of gender, males had a higher risk of secondhand smoke exposure compared to females (PR:1.3, 95%CI:1.257 ~ 1.323). Regarding educational level, individuals with a bachelor’s degree or higher had a lower risk of secondhand smoke exposure compared to those with a college degree or below (PR:0.9, 95%CI:0.907 ~ 0.963), while there was no significant difference in secondhand smoke exposure rate for those with junior high school education or below compared to the reference group. In terms of socioeconomic status, levels 2 and 3 had no significant difference in secondhand smoke exposure rate compared to level 1, while level 4 (PR:0.9, 95%CI:0.864 ~ 0.999), level 5 (PR:0.9, 95%CI:0.802 ~ 0.931), level 6 (PR:0.8, 95%CI:0.754 ~ 0.888), and level 7 (PR:0.8, 95%CI:0.718 ~ 0.0.856) were all protective factors. It is noteworthy that there was no significant difference in secondhand smoke exposure rate among different levels of employment status, residence, and marital status.

### Predictive risk maps

**Fig. 2:**
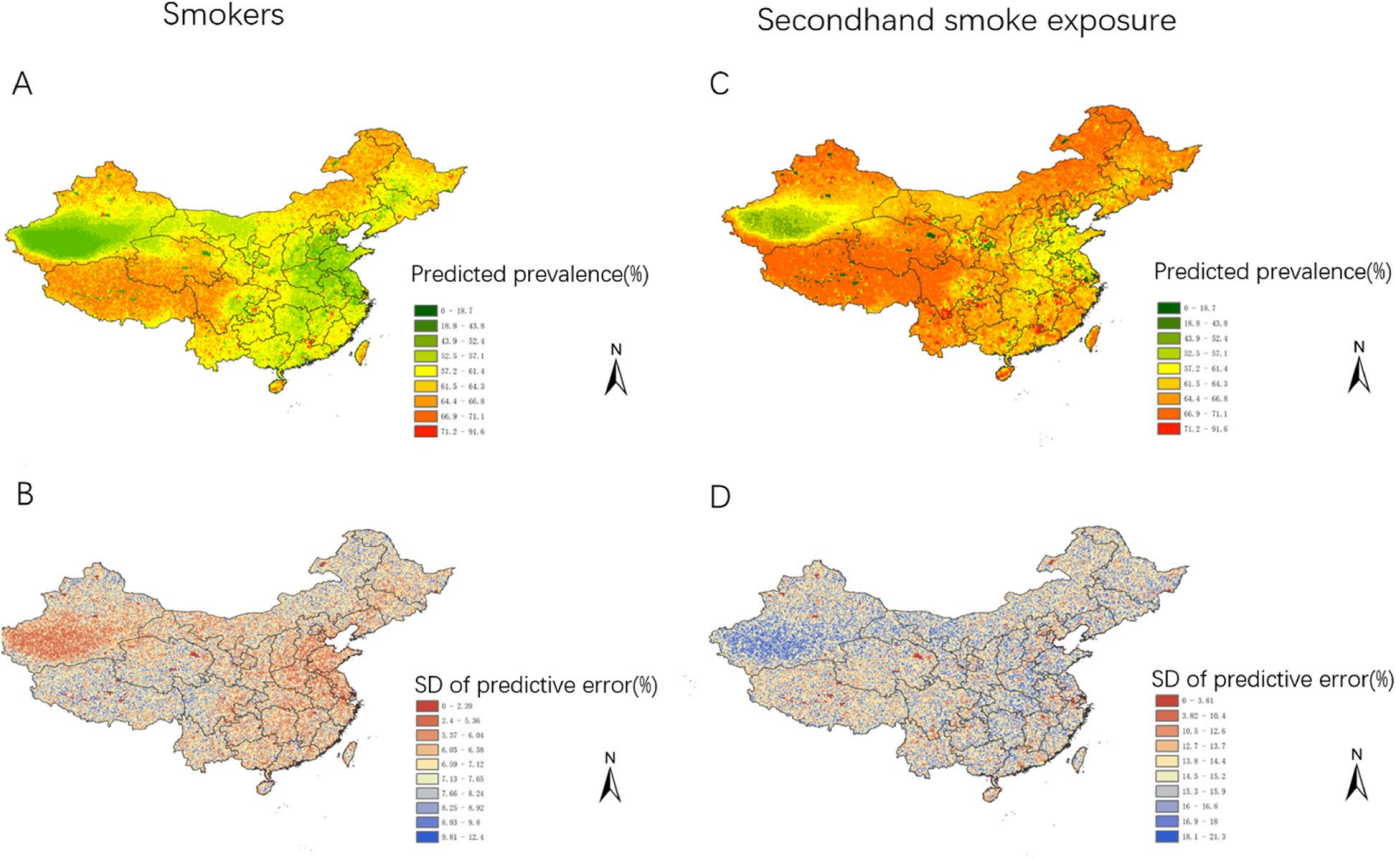
Model-based predictive risk maps

 The study delineates the specific locations and circumstances associated with smokers and secondhand smoke exposure across diverse regions. In this context, the designation of “Yes” is assigned to samples exhibiting both smokers and secondhand smoke exposure, whereas “No” is attributed to samples lacking these aforementioned conditions (Supplementary Fig. 4). The distribution of sampling points is predominantly concentrated in central and eastern regions of China, with a lower number of sampling points in western regions such as Tibet, Qinghai, and Xinjiang. Additionally, it can be observed that the sample size for smokers is significantly lower than that for secondhand smoke exposure. Figure 2A-B and C-D show the unadjusted risk graphs of predicted prevalence and corresponding prediction errors for smokers and secondhand smoke exposure, respectively (Fig. 2). These figures primarily forecast a high prevalence of smokers in western and northeastern regions of China, as well as northern Xinjiang, with prevalence rates ranging from 15.3 to 46.2%. The prevalence rate in central regions is moderate, ranging from 13 to 15.2%, while southern Xinjiang and eastern regions have lower prevalence rates (< 13%). On the other hand, secondhand smoke exposure has a high prevalence in western and northeastern regions, with prevalence rates ranging from 64.4 to 91.6%, the moderate prevalence in eastern regions (57.2-64.3%), and lower prevalence rates in southern Xinjiang (< 57.1%).

### Estimates of the population-adjusted prevalence

Based on 2020 population density data from the Centre for International Geoscience Information Network[25] and projections of risk and spatial geography using results from Bayesian geostatistical models, we developed population-adjusted projections of smokers and secondhand smoke exposure rates based on provincial, prefecture-level, and rural areas. Population-adjusted disease rates encompass both the rates of smokers and the population exposure to secondhand smoke, adjusting for age structures, gender ratios, and economic conditions. In light of this, smoking rates could be standardized through population adjustment to control for differences in demographic factors across diverse populations. This standardization enhances comparability between various groups, ultimately contributing to a more accurate understanding of the disparities in smoking rates among different populations. This adjustment aims to eliminate or diminish variations between different demographic groups, ensuring a more accurate and equitable comparison of smoking rates. Typically, distinct demographic groups may exhibit variations in age distribution, gender proportions, and economic status, which could impact smoking rates. Through population adjustment, smoking rates can be standardized, enhancing comparability across different groups.

**Fig. 3:**
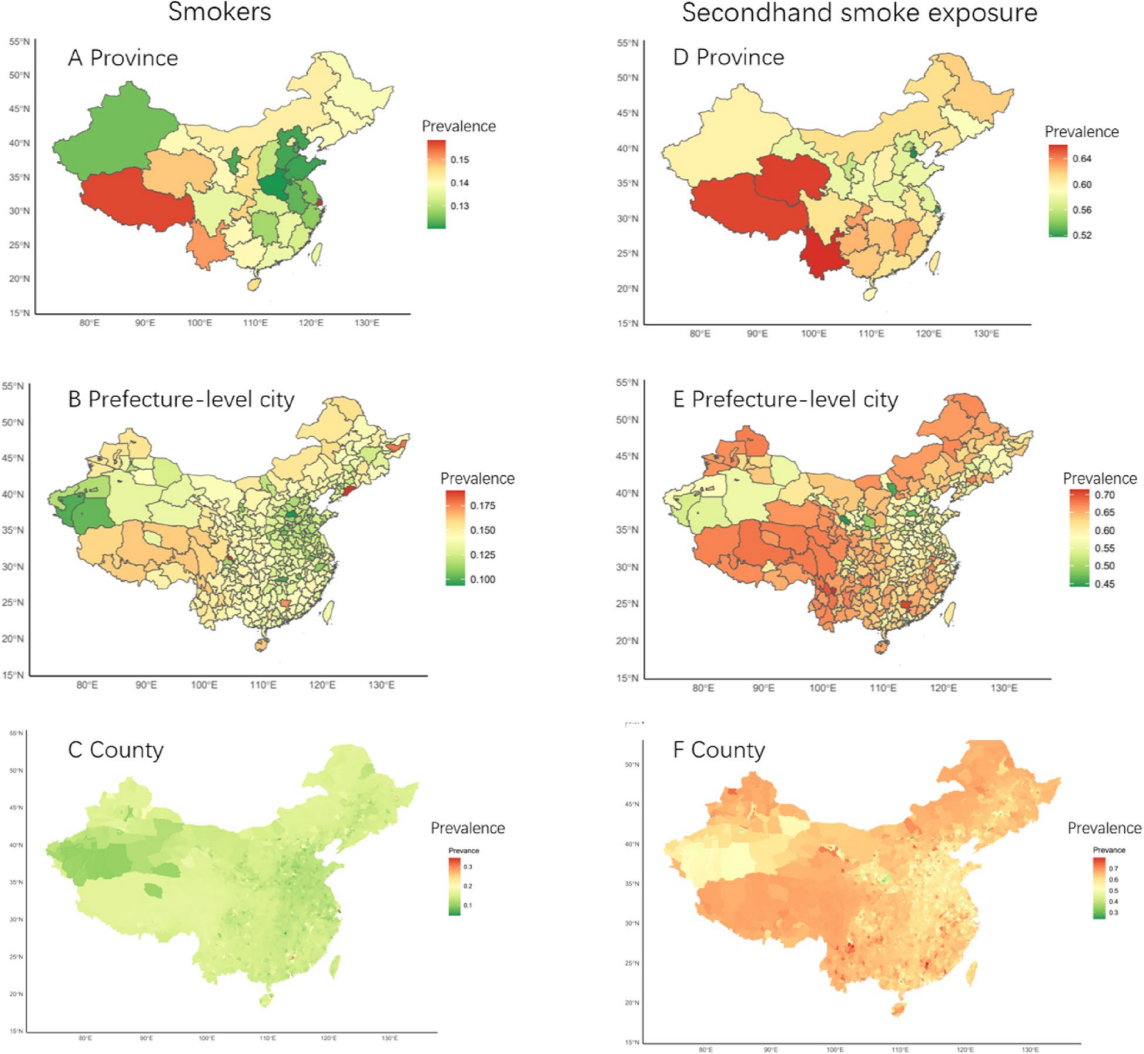
Population adjusted map

 As shown in Fig. 3A and C, the prevalence of smokers is highest in Tibet Autonomous Region, Shanghai Municipality, and Yunnan Province, exceeding 15%; whereas the lowest rates of smokers are found in Eastern provinces such as Xinjiang Uygur Autonomous Region, Ningxia Hui Autonomous Region, Henan, Tianjin, Hebei, and Shandong, where the rates are below 13%. Among prefecture-level cities, Dan Dong City in Liaoning Province, De Yang City in Sichuan Province, Shuang Ya Shan City in Heilongjiang Province, and Shaoguan City in Guangdong Province have higher rates of smokers (> 17.5%); whereas Kashgar, Hotan, Aksu, and Kizilsu Kirghiz Autonomous Prefecture in Xinjiang Province, Changsha City in Hunan Province, and most of the eastern cities have lower rates of smokers (< 10%). In the county-level map, it is clear that smoking rates are generally lower in county-level areas (< 15%), especially in coastal areas (< 5%). To differentiate it from other areas, we have used a dark green alternative here, representing a generally lower level.

According to Fig. 3D and F, the prevalence of secondhand smoke exposure is high (> 64%) in the Tibet Autonomous Region, Qinghai Province, and Yunnan Province, while Tianjin and Shanghai have lower secondhand smoke exposure rates (< 52%). Among prefecture-level areas, the secondhand smoke exposure rates are generally higher (> 65%) in the western region, northern Xinjiang, northeastern region, and southern coastal areas. Only Lanzhou, Qingyang, and Pingliang cities in Gansu Province, Hohhot in the Inner Mongolia Autonomous Region, and Xingtai City in Hebei Province have lower secondhand smoke exposure rates (< 50%). The predicted map of country-level shows that secondhand smoke exposure rates are generally higher (> 60%) nationwide. Additionally, we have included supplementary analytical data for each province, prefecture-level city, and county in an appendix folder to enhance the explanatory clarity of our results. These additional data aim to complement and provide further elucidation to Fig. 3, facilitating a clearer presentation of the smokers and secondhand smoke exposure situations across the three different hierarchical levels.

## Discussion

By compiling georeferenced measurement data and utilizing a Bayesian geostatistical model based on multiple environmental and economic factors, we were able to calculate model-based high-resolution estimates of smoking prevalence risk within the scope of China. Furthermore, we analyzed the protective and harmful effects that exposure factors related to smokers and exposure to secondhand smoke may have on two outcome variables. The findings obtained through the use of this Bayesian geostatistical model and the analysis of exposure factor effects are crucial for prioritizing regions, providing smoking cessation services, and developing smoking cessation intervention guidelines. While studies have generated some relevant maps, such as the 2018 China GATS, which gathered provincial-level data and reported smoking rates regionally, and the China Center for Disease Control and Prevention conducted studies and disseminated provincial-level adolescent smoking data, these existing studies have not provided explicit geographical distribution maps of tobacco use and maps illustrating the risk of smoking prevalence utilizing Bayesian geographical statistical models. Moreover, the 2018 China General Agreement on Trade in Services and the forecast chart produced by the Chinese Center for Disease Control and Prevention only reflect the differences between provinces. Therefore, our research will fill these gaps.

Our analysis of exposure factors showed that the number of smokers among the study population was far less than the number of non-smokers, whereas the number of individuals exposed to secondhand smoke was greater than those who were not exposed. In multivariate analysis, the highest risk factor for smokers was the male gender, with a significantly higher smoking rate compared to females. This may be attributed to Chinese culture (e.g., drinking culture, giving cigarettes as a greeting among men), customs (e.g., giving cigarettes as gifts during festivals), male attitudes, and lifestyle [12, 26]. Additionally, having a Bachelor’s degree or higher was also a significant protective factor, indicating that higher education levels could reduce the occurrence of smokers, which is closely related to this population’s knowledge and awareness [16]. The most significant risk factor for exposure to secondhand smoke was the age group between 18 and 59 years, possibly because most of these individuals belong to the working population, and their work environment has a considerable impact on exposure to secondhand smoke. Social status was the most significant protective factor. It is noteworthy that as the level of social status increases, the PR value decreases, indicating that individuals with higher social status have a lower likelihood of exposure to secondhand smoke. A systematic review found that isolated individuals are more likely to smoke than popular ones[27], indicating that higher social status may represent better work and living environments and social circles (more popular), resulting in a lower probability of smoking and exposure to secondhand smoke.

The Bayesian geostatistical model identified two environmental predictor factors, namely, nighttime light index and PM2.5, which are positively associated with the prevalence of smoking. Higher values of the nighttime light index and PM2.5 reflect higher economic and developmental status, both of which are negatively correlated with the prevalence of tobacco consumption. Regions with higher economic status and more rapid development tend to exhibit lower rates of smokers and secondhand smoke exposure. Generally, rural areas have lower economic status and less development, which results in a higher prevalence of smoking among the rural population. These findings are by the predictive maps we created as well as previous studies[28]. Furthermore, the distinct economic and environmental characteristics reflected by the two factors can more effectively demonstrate geographical features, and aid in analyzing the differences in the prevalence of smokers and secondhand smoke exposure among different types of regions.

The predictive risk map we have generated indicates that smokers and secondhand smoke exposure are predominantly high-risk in the western and northeastern regions of China. The predictive map also reveals that smokers and secondhand smoke exposure are more common in the central and southern regions than in the eastern region, possibly due to the large-scale production and consumption of tobacco in these areas. In China, tobacco production mainly occurs in Yunnan and Guizhou provinces, and over 70% of tobacco leaves are estimated to be produced in the less-developed provinces in the central and western regions of the country. Rural households in southwestern China spend an average of 6.5% of their total income on tobacco[29]. This is consistent with our model’s incorporation of the variable of nighttime light intensity, which reflects a higher level of economic development and a higher prevalence of smoking.

We predicted the prevalence of tobacco use across three levels of regions: province, city, and rural areas. First, in the predicted prevalence map of smokers adjusted by population, Tibet Autonomous Region and Shanghai had high rates of tobacco use. Notably, the production area of Chinese tobacco does not include Beijing, Shanghai, Tianjin, and Tibet [29], yet these regions still showed higher rates of tobacco use. Moreover, previous studies have confirmed that Shanghai’s smoke-free policy has become a model that has been widely promoted nationwide[30], which seemingly conflicts with our findings. Thus, we conducted additional analyses of smoking behaviors among people with an IP address in Shanghai and found that traditional tobacco use in Shanghai was indeed lower than in other provinces but the use of electronic cigarettes was much higher. This indicates that electronic cigarette use has become a new significant obstacle to the implementation and promotion of smoking cessation interventions under the current situation where traditional tobacco is under widespread control, which merits attention in China and globally. Second, the prevalence of smokers in urban areas is generally at a medium to low level, with only a few cities showing higher rates. China’s urban areas have had a unique opportunity since the establishment of the Global Health Institute-China Tobacco Control Partnership (GHI-CTP) in 2008, which focuses on changing social norms of tobacco use in Chinese cities through the “bottom-up” approach[31]. Additionally, approximately half of China’s cities are reported to have implemented smoke-free policies[31], which significantly promotes their pivotal role in smoking cessation interventions and potentially changes the pattern of smoking cessation actions in China. Finally, we found that the prevalence of smokers in rural areas is generally higher than in provinces and cities. Previous studies on tobacco use in rural areas have also demonstrated that there may be more diverse ethnic, cultural, and economic differences in rural areas than in provinces and cities, which play crucial roles in the prevalence and behavior of tobacco use[32]. Thus, providing cultural assistance and addressing significant socioeconomic determinants in rural areas will contribute to minimizing the differences in tobacco use and smoking behavior in China, thereby promoting public health.

Unlike smokers, exposure to secondhand smoke is more common in China, which leads to a higher incidence of diseases and heavier economic burdens. Therefore, it may become a critical issue that needs to be addressed by the state.

In the epidemiological prevalence prediction map of secondhand smoke exposure adjusted for population, Yunnan, Tibet, and Qinghai provinces have the highest secondhand smoke exposure rates, which may be attributed to the local environmental climate and cultural customs. Furthermore, all three provinces are situated in the southwestern region, and some studies have shown that smoking is more prevalent among males in this area[32]. Additionally, the southwestern region of China is relatively underdeveloped, and tobacco cultivation is widespread, which may also contribute to the higher secondhand smoke exposure rates[33]. Shanghai and Beijing have relatively low secondhand smoke exposure rates, as both cities are economic and cultural centers, and have implemented more stringent smoke-free policies. In Shanghai, a comprehensive smoke-free household policy has been widely promoted, and more and more families have become smoke-free, indirectly reducing secondhand smoke exposure rates[30]. However, the prevalence of smokers in Shanghai remains high, indicating that even with the complete promotion of smoke-free policies, the popularity of electronic cigarettes has not been effectively controlled, which can exacerbate the secondhand smoke exposure situation. The country needs to develop more intervention measures and policies to comprehensively control electronic cigarettes to truly improve China’s smoking problem. Secondly, the secondhand smoke exposure rates of the majority of cities at the prefectural level are relatively high, with only some cities in the eastern parts and southern Xinjiang having lower rates. From the predicted map of smokers, it can be seen that the prevalence rates of smokers in these two regions are also lower than in other regions, which results in lower secondhand smoke exposure rates, possibly due to the correlation with their levels of economic development. Finally, our study found that secondhand smoke exposure rates in rural areas are generally higher. With 70% of China’s population living in rural areas, the large base has significantly increased the secondhand smoke exposure rate among rural populations compared to urban populations [5, 33]. Moreover, many rural areas rely on tobacco cultivation as the foundation of their local economy, making it difficult to avoid the problem of secondhand smoke exposure [33].

Despite China’s current vigorous promotion and implementation of smoke-free policies, effectively contributing to a reduction in smoking rates, inherent limitations persist, resulting in a relatively gradual decline in smoking prevalence. Presently, smoke-free policies in China are predominantly promoted and enforced in urban areas, with provincial capitals showing a higher likelihood of adopting such regulations compared to other cities. Among the 21 cities that have enacted smoke-free laws, 9 prohibit smoking in all indoor workplaces, indoor public spaces (restaurants, bars, health facilities, government buildings, and schools), and public transportation. More than half of the smoke-free laws still allow designated indoor smoking areas. Smoke-free laws explicitly prohibiting the use of electronic cigarettes in smoke-free zones are implemented in only two cities (Nanning and Hangzhou)[34]. As of 2012, over three-quarters of the surveyed individuals reported smoking in bars, with more than two-thirds smoking in restaurants, and over half engaging in smoking within indoor workplaces[35]. Furthermore, the substantial size of China’s tobacco market poses a significant obstacle to tobacco control due to the economic benefits derived from tobacco production and sales. Additionally, the complete control exercised by the China National Tobacco Corporation (CNTC) and the State Tobacco Monopoly Administration (STMA) over tobacco production, sales, and import-export operations is considered a barrier to the implementation of the Framework Convention on Tobacco Control (FCTC)[36]. Therefore, China needs to make further efforts to address these limitations to achieve more effective tobacco control.

We have conducted an extensive literature review and observed that China has developed a series of interventions and implemented pertinent policies to control the prevalence of tobacco[37–39]. Considering the prevailing situation of relatively scarce and unevenly distributed healthcare resources in China, further development of new preventive and control measures would entail a wasteful utilization of limited resources. Conversely, the development of scientific implementation strategies would significantly facilitate the effective realization of existing measures in light of Implementation Science[40, 41]. Specifically, researchers and policy-makers should initially analyze the barriers that hinder the implementation of these policies and interventions. Subsequently, corresponding implementation strategies should be tailored to eliminate these barriers. It is noteworthy that implementation strategies, in essence, are also interventions directed toward healthcare service providers rather than recipients. The purpose of such implementation strategies is to encourage healthcare professionals to adopt evidence-based practice in clinical and public health work, thereby enhancing the quality of healthcare services, as opposed to directly improving patient health outcomes through traditional interventions[42]. Therefore, we contend that the development of scientifically effective implementation strategies to promote the implementation of evidence-based tobacco control policy and interventions holds practical value and real-world significance.

Our study has mainly three main limitations. Firstly, the Bayesian statistical model we used had a lower AUC value of 0.658 for predicting smokers after model validation (AUC = 0.5 ~ 0.7 indicates a relatively lower predictive performance). Despite this, the variables included in our model are more scientifically robust, and the stability of the model was ensured through multiple validation approaches. Secondly, our sample was mainly collected from the central and eastern regions of China, with a relatively smaller number of samples from the western region, which may prevent the discovery of stronger associations in the multivariate analysis. However, it is worth noting that this helps us identify data-scarce areas and is useful in planning future study investigations. Thirdly, even though interpreting PM2.5 concentrations in the air as an indicator of urbanization is not impossible at coarse spatial resolutions, it is deemed unsuitable for predicting disease rates at higher spatial resolutions due to the considerable influence of atmospheric dynamics on air pollution metrics.

## Conclusion

In summary, we propose for the first time a model-based, high-resolution nationwide assessment of smoking risk and apply a rigorous Bayesian geostatistical model to help draw smoking prevalence prediction maps based on socioeconomic and environmental factors. These maps provide estimates of the geographic distribution of smoking, which will serve as strong evidence for the development and implementation of smoking cessation policies. Additionally, in the current context of the nation’s strong efforts to control traditional tobacco, the increasing use of electronic cigarettes undoubtedly poses a significant challenge to national smoking intervention. Therefore, the prevalence of electronic cigarette use and effective prevention and control measures will also become a focus of our future studies.

### Supplementary Information

**Supplementary Material 1.**

## Data Availability

All data generated or analyzed during this study are with the corresponding author. She is available to answer any questions about the datasets. Persons who have made outstanding contributions or assisted in this study may apply for the use of the data only after submitting the study hypothesis and signing a data confidentiality agreement. There is no fee for the data opening plan. Publication of the study results will include processed data only, and personal information will remain anonymous.
