# Supplementary material for: Risk profiling of tobacco epidemic and estimated number of smokers living in China: a cross-sectional study based on PBICR
Source: BMC Public Health. 2024 Aug 15;24:2219. doi: 10.1186/s12889-024-18559-x (PMC11325620; doi:10.1186/s12889-024-18559-x)
Supplement: Supplementary file 1 — Supplementary Material 1. [file 12889_2024_18559_MOESM1_ESM.docx]

Supplementary Material

**Risk profiling of tobacco epidemic and estimated number of smokers living in China: A cross-sectional study based on PBICR**

**Siyuan Liu, PhD^1#^, Haozheng Zhou, BS^1#^ , Na Ren, PhD^3,4^, Wenjun He, PhD^1^, Jiao Yang, BS^2^ , Xuanhao Yin , BS^1^ , Sufeila·Shalayiding, BS^2^ , Yan Zhou, BS^4^, Xinyi Rao, BS^2^ , Nuofan Zhang, BS^2^ , Man Xiong, BS^2^ , Yueying Wang BS^2^, Wenfu Yang, BS^2^ , Yibo Wu, PhD^5*^, Jiangyun Chen, PhD^2*^**

^1^School of Public Health, Southern Medical University, Guangzhou510515, China;

^2^School of Health Management, Southern Medical University, Guangzhou510515, China;

^3^Institute of Chinese Medical Sciences, University of Macau, Macau, China;

^4^Operation Management Department, Zhuhai People's Hospital (Zhuhai Hospital Affiliated with Jinan University), Zhuhai, China;

^5^School of Public Heath, Peking University, Beijing100091, China;

^#^These authors contributed equally to this work.

^*^Corresponding author.

Jiangyun Chen

Tel:18588220304

E-mail: [cjy112@i.smu.edu.cn](mailto:cjy112@i.smu.edu.cn)

School of Public Health, Southern Medical University, Guangzhou510515, China.

Or Yibo Wu

Tel:18810169630

E-mail: bjumuwuyibo@outlook.com

School of Public Heath, Peking University, Beijing100091, China.

**Statistical analysis**

We investigated the impact of various socio-demographic characteristics on both smokers and secondhand smoke exposure. Firstly, we conducted a univariate analysis to present the results of the impact of each characteristic. Secondly, we performed a multivariate retrospective analysis using the log-binomial link function to assess the combined effects of multiple characteristics on smoking behavior. The prevalence ratios were presented using forest plots to provide a clear visualization of the relationships between the different factors and smoking behavior. A prevalence ratio greater than 1 indicates the exposure factor is a risk factor, while a prevalence ratio less than 1 indicates a protective factor.

The data cleaning and statistical analyses of this study were conducted using SAS V9.4 and R software. All tests were two-tailed with P < 0.05 indicating statistically significant differences.

**Model validation**

The model validation showed that the AUC (Supplementary Figure 3) for smokers and secondhand smoke exposure were 0.658 and 0.708, respectively, indicating that the overall predictive performance for smokers is still acceptable, while the overall predictive performance for secondhand smoke exposure is better.

**Univariates analysis**

The basic characteristics of smokers and smoke-exposed individuals are presented in the univariate analysis (Supplementary Table 5). Overall, among the 21,810 study subjects, the number of smokers reached 3,219 people, accounting for 14.8% of the total, while the number of smoke-exposed individuals reached 11,415, accounting for 52.3% of the total. Both outcome variables of smokers and secondhand smoke exposure showed statistical differences (all P<0.01) among age, gender, education level, family per capita monthly income, occupational characteristics, place of residence, marital status, and social status. In addition, no statistical significance was found between ethnicity, religious belief, smokers, and secondhand smoke exposure (all P>0.05).

Table and figure legends

**Supplementary Table 1** Outcome Variable Description

**Supplementary Table 2** Covariate Variable Description

**Supplementary Table 3** Posterior summaries(median and 95% Bayesian credible interval) of the geostatistical mode

**Supplementary Table 4** Variable selection in Bayesian model constructionl

**Supplementary Table 5** Correlation analysis of basic characteristics between smokers and those exposed to secondhand smoke

**Supplementary Figure 1** Data search and selection

**Supplementary Figure 2** Correlation analysis is used to explain the collinearity of Bayesian geostatistical model construction

**Supplementary Figure 3** Results of the ROC curve

**Supplementary Figure 4** Survey Location and observed status over the study region

**Supplementary Table 1 Outcome variable description**

| Outcome Variable | Definition or code |
| --- | --- |
| Smokers | 1=Vaping/smoking cigarettes/both; 0=Never smoked/Quit smoking |
| Secondhand smoke exposure | 1=Individuals have a smoking habit in my immediate environment and frequently smoke around me;  0=Individuals without smoking habit or Individuals have a smoking habit in my immediate environment but don't smoke around me |

**Supplementary Table 2 Covariate variable description.**

| Covariates name | Definition or code |
| --- | --- |
| Age | 1=12~17; 2=18~59; 3=≥60. |
| Gender | 1=male; 2=female. |
| Highest Education | 1=Junior high school and below; 2= College and below; 3= Undergraduate and above |
| Monthly family income per capita | 1=<3000; 2=3000~6000; 3=>6000. |
| Ethnicity | 1=Han; 2=Others. |
| Religious | 0=No; 1=YES. |
| Work status | 1=Be employed; 2=Student; 3=Retirement; 4, 5, 6=Unemployed. |
| Permanent Residence | 1=Urban; 2=Rural. |
| Marital Status | 1=Unmarried; 2=Married; 3=Divorce; 4=Bereaved spouse. |
| Social Status | The social status was divided into seven levels, and the continuous variable was converted into the type variable, in which the higher the level, the higher the social status |

**Supplementary Table 3** **Posterior summaries(median and 95% Bayesian credible interval) of the geostatistical model**

| Outcome | Estimate |
| --- | --- |
| **Smokers** |  |
| Night Light | -0.003(-0.006;0.000) |
| PM2.5 | -0.005(-0.009;-0.001) |
| Range(km) | 12.8(11.90;13.80) |
| Spatial variance ($\sigma_{sp}^{2}$) | 0.839(0.671;1.076) |
| Non-Spatial variance($\sigma_{nonsp}^{2}$) | 0.009(0.008;0.010) |
| **Secondhand smoke exposure** |  |
| Night Light | -0.005(-0.008;-0.003) |
| PM2.5 | -0.006(-0.010;-0.001) |
| Range(km) | 17.2(12.00;21.96) |
| Spatial variance ($\sigma_{sp}^{2}$) | 1.028(0.854;1.234) |
| Non-Spatial variance($\sigma_{nonsp}^{2}$) | 0.006(0.004;0.013) |

**Supplementary Table 4 Variable selection in Bayesian model construction**

| Smoke condition | Models | DIC |
| --- | --- | --- |
| Smokers | night light | 17513 |
|  | ^a^night light + PM2.5 | 17509.2 |
|  | night light + PM2.5+ Elevation | 17509.72 |
|  | night light + PM2.5+ Elevation +terrestrial | 17509.73 |
|  | night light + PM2.5+ Elevation +terrestrial+LST | 17509.85 |
|  | night light+ PM2.5 + Elevation +terrestrial+LST+HII | 17510.17 |
|  | night light + PM2.5+ Elevation +terrestrial+LST+HII+IMR | 17509.76 |
|  | night light + PM2.5+ Elevation +terrestrial+LST+HII+IMR+HBSE | 17512.7 |
| Secondhand smoke exposure | night light | 27865.3 |
|  | ^a^night light + PM2.5 | 27862.73 |
|  | night light + PM2.5+ Elevation | 27866.84 |
|  | night light + PM2.5+ Elevation +terrestrial | 27865.01 |
|  | night light + PM2.5+ Elevation +terrestrial+LST | 27865.71 |
|  | night light+ PM2.5 + Elevation +terrestrial+LST+HII | 27866.92 |
|  | night light + PM2.5+ Elevation +terrestrial+LST+HII+IMR | 27867.3 |
|  | night light + PM2.5+ Elevation +terrestrial+LST+HII+IMR+HBSE | 27866.84 |

Notes: ^a^ Bayesian model selected by DIC.

**Supplementary Table 5** **Correlation analysis of basic characteristics between smokers and those exposed to secondhand smoke.**

| Variables（%） | levels | All participants | Smokers | | | Secondhand smoke exposure | | |
| --- | --- | --- | --- | --- | --- | --- | --- | --- |
|  |  |  | NO | YES | *p* | NO | YES | *p* |
| N |  | 21810 (100) | 18591(85.2) | 3219 (14.8) |  | 10395(47.7) | 11415(52.3) |  |
| Age | 12~17 | 2053(9.4) | 1942 (10.4) | 111 (3.4) | <0.001 | 1195 (11.5) | 858 (7.5) | <0.001 |
|  | 18~59 | 15580(71.4) | 13197 (71.0) | 2383 (74.0) |  | 6816 (65.6) | 8764 (76.8) |  |
|  | ≥60 | 4177(19.2) | 3452 (18.6) | 725 (22.5) |  | 2384 (23.0) | 1793 (15.7) |  |
| Gender | Female | 10914(50.0) | 10539 (56.7) | 375 (11.6) | <0.001 | 5887 (56.6) | 5027 (44.0) | <0.001 |
|  | Male | 10896(50.0) | 8052 (43.3) | 2844 (88.4) |  | 4508 (43.4) | 6388 (56.0) |  |
| Highest Education | Junior high school and below | 6954(31.9) | 5732 (30.8) | 1222 (38.0) | <0.001 | 3447 (33.2) | 3507 (30.7) | <0.001 |
|  | College and below | 7651(35.1) | 6420 (34.5) | 1231 (38.2) |  | 3518 (33.8) | 4133 (36.2) |  |
|  | Undergraduate and above | 7205(33.0) | 6439 (34.6) | 766 (23.8) |  | 3430 (33.0) | 3775 (33.1) |  |
| Monthly family income per capita | <3000 | 7191(33.0) | 6032 (32.4) | 1159 (36.0) | <0.001 | 3261 (31.4) | 3930 (34.4) | <0.001 |
|  | 3000~6000 | 8979(41.2) | 7739 (41.6) | 1239 (38.5) |  | 4376 (42.1) | 4602 (40.3) |  |
|  | >6000 | 5641(25.9) | 4820 (26.0) | 821 (25.5) |  | 2758 (26.5) | 2758 (24.2) |  |
| Ethnicity | Han | 19837(91.1) | 16967 (91.3) | 2906 (90.3) | 0.074 | 9498 (91.4) | 10375 (90.9) | 0.221 |
|  | Others | 1937(8.9) | 1624 (8.7) | 313 (9.7) |  | 897 (8.6) | 1040 (9.1) |  |
| Religious | NO | 20959(96.0) | 17883 (96.2) | 3076 (95.6) | 0.096 | 9994 (96.1) | 10965 (96.1) | 0.774 |
|  | YES | 851(3.9) | 708 (3.8) | 143 (4.4) |  | 401 (3.9) | 450 (3.9) |  |
| Work status | Student | 6534(30.0) | 6109 (32.9) | 425 (13.2) | <0.001 | 3172 (30.5) | 3362 (29.5) | <0.001 |
|  | Be employed | 7577(34.7) | 6187 (33.3) | 1390 (43.2) |  | 3354 (32.3) | 4223 (37.0) |  |
|  | Unemployed | 4963(22.8) | 4043 (21.7) | 920 (28.6) |  | 2331 (22.4) | 2632 (23.1) |  |
|  | Retirement | 2736(12.5) | 2252 (12.1) | 484 (15.0) |  | 1538 (14.8) | 1198 (10.5) |  |
| Permanent Residence | Urban | 15111(69.3) | 13035 (70.1) | 2076 (64.5) | <0.001 | 7288 (70.1) | 7823 (68.5) | 0.012 |
|  | Rural | 6699(30.7) | 5556 (29.9) | 1143 (35.5) |  | 3107 (29.9) | 3592 (31.5) |  |
| Marital Status | Married | 12397(56.8) | 10156 (56.6) | 2241 (69.6) | <0.001 | 5904 (56.8) | 6493 (56.9) | <0.001 |
|  | Unmarried | 8442(38.7) | 7650 (41.1) | 792 (24.6) |  | 4015 (38.6) | 4427 (38.8) |  |
|  | Bereaved spouse | 570(2.6) | 488 (2.6) | 82 (2.5) |  | 312 (3.0) | 258 (2.3) |  |
|  | Divorce | 401(18.3) | 297 (1.6) | 104 (3.2) |  | 164 (1.6) | 237 (2.0) |  |
| Social Status | Level 1 | 431(2.0) | 337 (1.8) | 94 (2.9) | <0.001 | 163 (1.6) | 268 (2.3) | 0.001 |
|  | Level 2 | 1071(4.9) | 868 (4.7) | 203 (6.3) |  | 416 (4.0) | 655 (5.7) |  |
|  | Level 3 | 3483(16.0) | 2915 (15.7) | 568 (17.6) |  | 1518 (14.6) | 1965 (17.2) |  |
|  | Level 4 | 7540(34.6) | 6529 (35.1) | 1011 (31.4) |  | 3504 (33.7) | 4036 (35.4) |  |
|  | Level 5 | 5555(25.5) | 4785 (25.7) | 770 (23.9) |  | 2793 (26.9) | 2762 (24.2) |  |
|  | Level 6 | 2250(10.3) | 1929 (10.4)) | 321 (10.0) |  | 1199 (11.5) | 1051 (9.2) |  |
|  | Level 7 | 1480(6.8) | 1228 (6.6) | 252 (7.8) |  | 802 (7.7) | 678 (5.9) |  |

Notes: Values were presented as n (%).


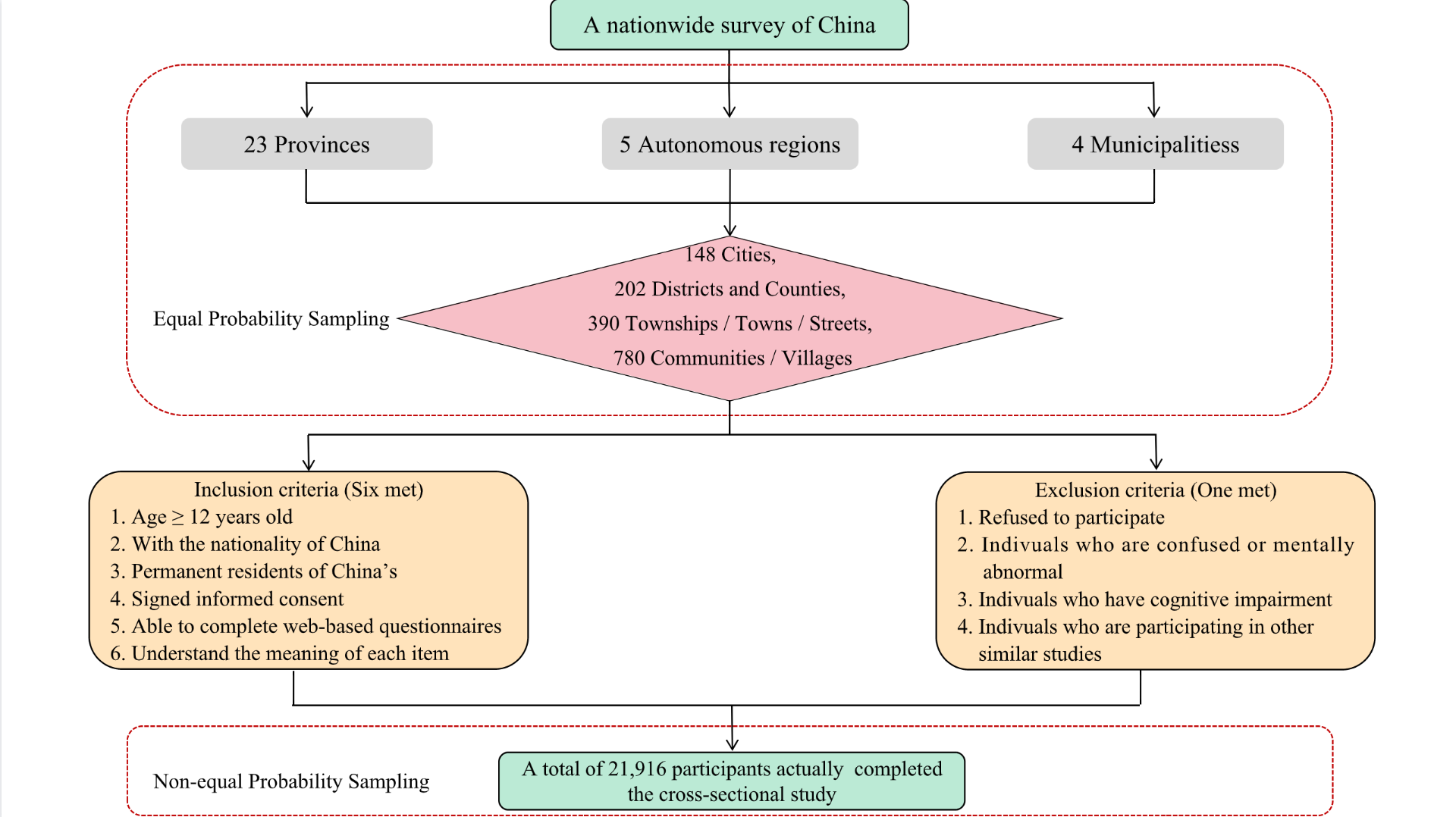


**Supplementary Figure 1 Data search and selection**


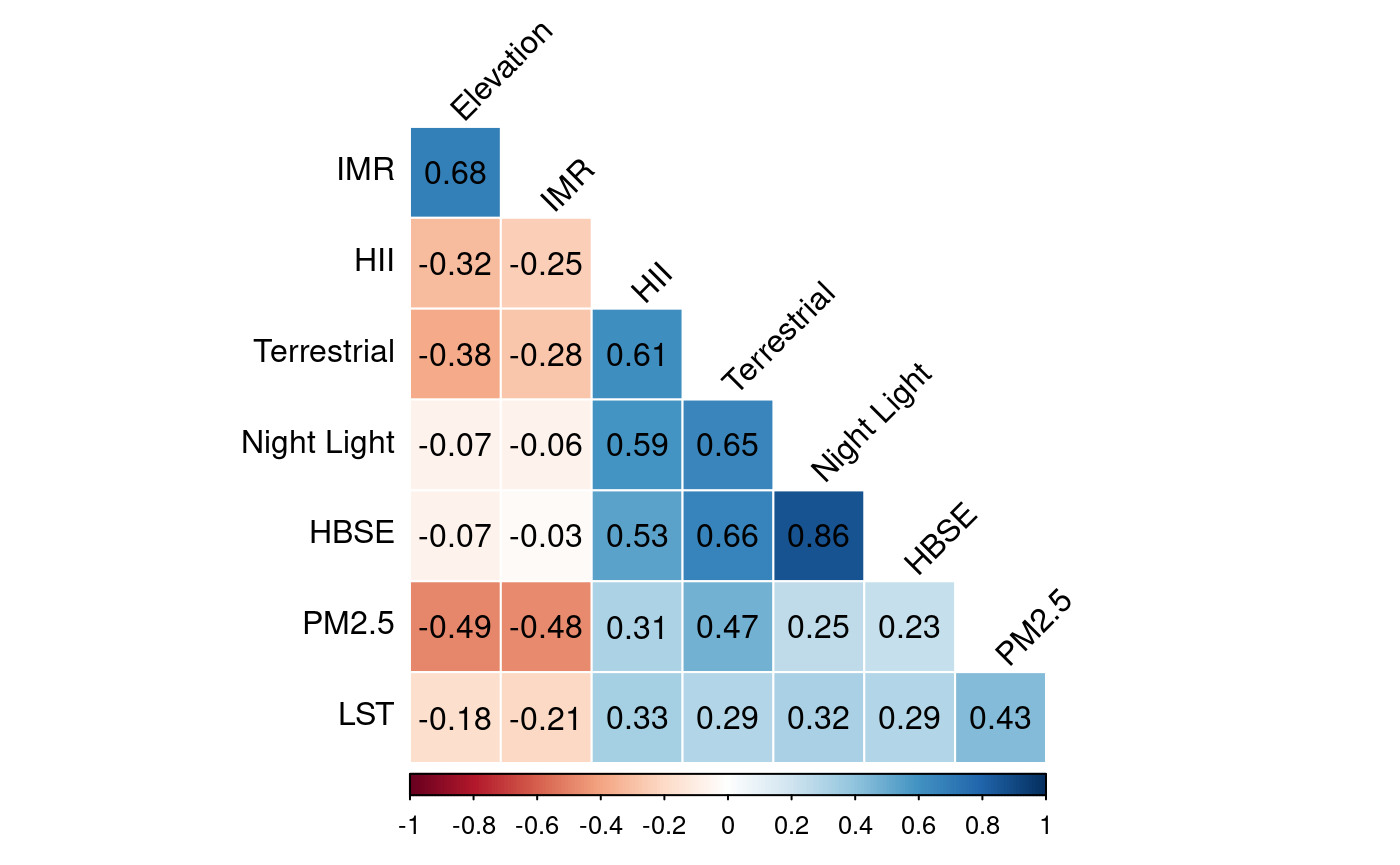


**Supplementary Figure 2 Correlation analysis is used to explain the collinearity of Bayesian geostatistical model construction.**


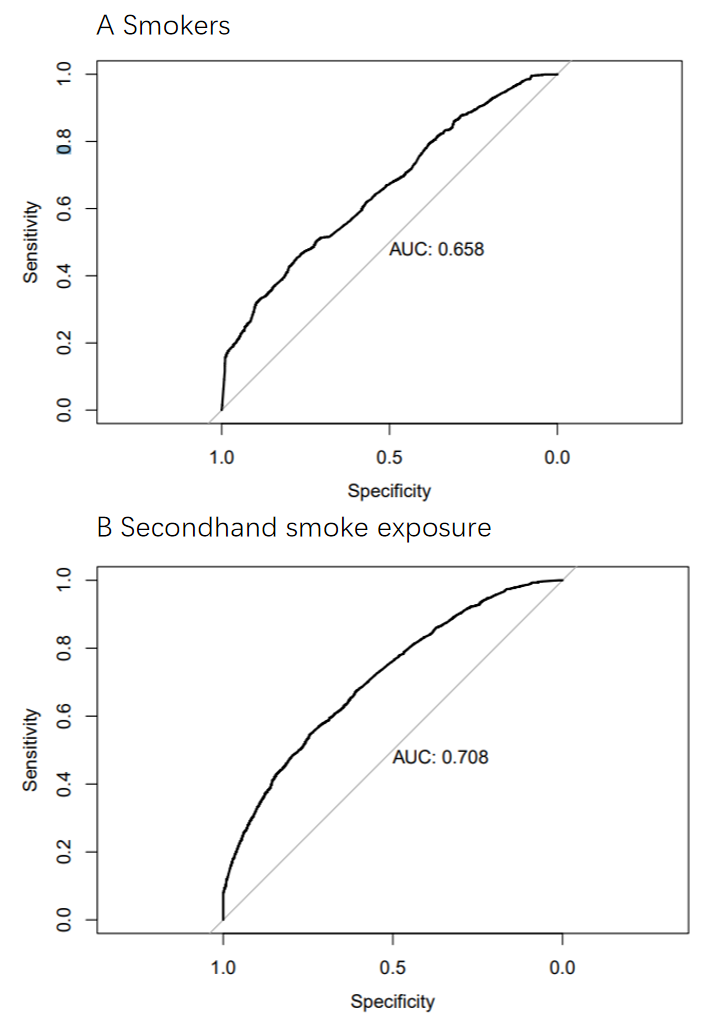


**Supplementary Figure 3 Results of the ROC curve**


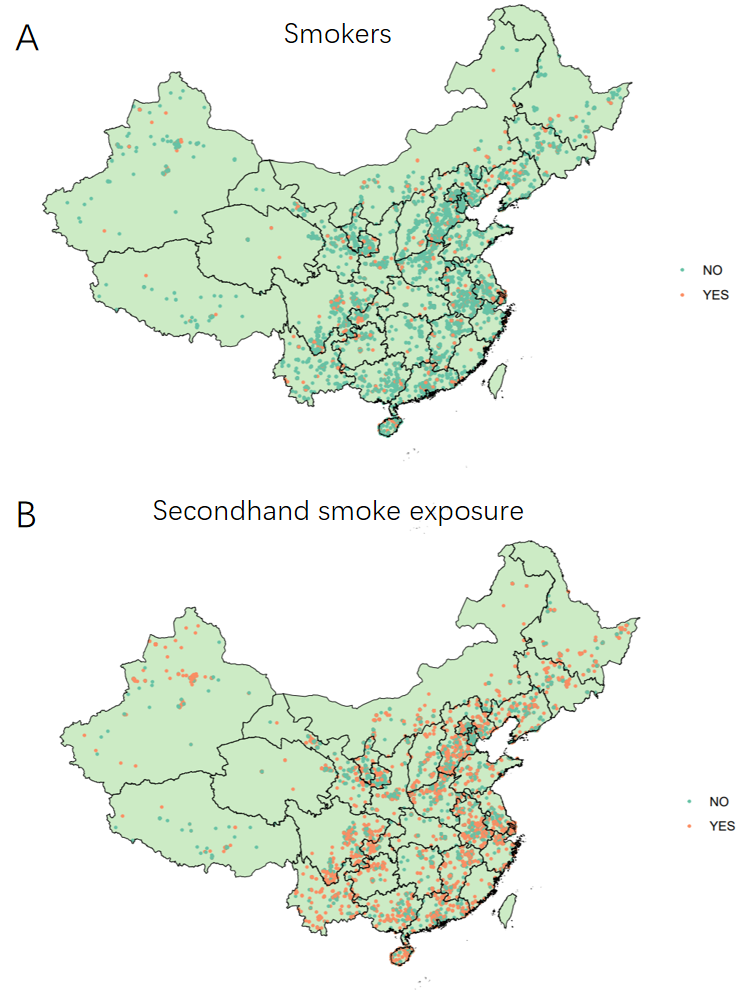


**Supplementary Figure 4 Survey Location and observed status over the study region**
